# Durability Performance of Self-Compacting Concrete Containing Crumb Rubber, Fly Ash and Calcium Carbide Waste

**DOI:** 10.3390/ma15020488

**Published:** 2022-01-09

**Authors:** Sylvia E. Kelechi, Musa Adamu, Abubakar Mohammed, Yasser E. Ibrahim, Ifeyinwa I. Obianyo

**Affiliations:** 1 Department of Mechanical and Civil Engineering, Purdue University Northwest, Hammond, IN 46323, USA; skelechi@pnw.edu; 2 Department of Civil Engineering, Bayero University, P.M.B 3011, Kano 700006, Nigeria; amohammed.civ@buk.edu.ng; 3 Engineering Management Department, College of Engineering, Prince Sultan University, Riyadh 11586, Saudi Arabia; 4 Department of Materials Science and Engineering, African University of Science and Technology, Abuja 900109, Nigeria; iobianyo@aust.edu.ng

**Keywords:** calcium carbide residue, crumb rubber, fly ash, self-compacting concrete, acid attack, salt attack

## Abstract

Waste tire disposal continues to pose a threat to the environment due to its non-biodegradable nature. Therefore, some means of managing waste tires include grinding them to crumb rubber (CR) sizes and using them as a partial replacement to fine aggregate in concrete. However, the use of CR has a series of advantages, but its major disadvantage is strength reduction. This leads to the utilization of calcium carbide waste (CCW) to mitigate the negative effect of CR in self-compacting concrete (SCC). This study investigates the durability properties of SCC containing CR modified using fly ash and CCW. The durability properties considered are water absorption, acid attack, salt resistance, and elevated temperature of the mixes. The experiment was conducted for mixes with no-fly ash content and their replica mixes containing fly ash to replace 40% of the cement. In the mixes, CR was used to partially replace fine aggregate in proportions of 0%, 10%, and 20% by volume, and CCW was used as a partial replacement to cement at 0%, 5%, and 10% by volume. The results indicate that the mixes containing fly ash had higher resistance to acid (H_2_SO_4_) and salt (MgSO_4_), with up to 23% resistance observed when compared to the mix containing no fly ash. In addition, resistance to acid attack decreased with the increase in the replacement of fine aggregate with CR. The same principle applied to the salt attack scenario, although the rate was more rapid with the acid than the salt. The results obtained from heating indicate that the weight loss was reduced slightly with the increase in CCW, and was increased with the increase in CR and temperature. Similarly, the compressive strength was observed to slightly increase at room temperature (27 °C) and the greatest loss in compressive strength was observed between the temperature of 300 and 400 °C. However, highest water absorption, of 2.83%, was observed in the mix containing 20% CR, and 0% CCW, while the lowest water absorption, of 1.68%, was found in the mix with 0% CR, 40% fly ash, and 10% CCW. In conclusion, fly ash is recommended for concrete structures immersed in water, acid, or salt in sulphate- and magnesium-prone areas; conversely, fly ash and CR reduce the resistance of SCC to heat beyond 200 °C.

## 1. Introduction

Environmental pollution causes a great danger to human health as well as contributing to the risk of the climate change. Efficient waste management has become increasingly essential since the industrial revolution. In the recent past, researchers have focused on the valorization of waste materials by turning them into wealth [1]. These waste materials could be recycled and reused for the development of other useful materials [2]. Cement is one of the most utilized construction materials due to its availability, but its production seriously affects environmental sustainability through the emission of high amounts of greenhouse gases such as CO_2_. The use of waste materials and by-products that have cementitious and Pozzolanic properties will significantly reduce the demand for cement in the construction industry and hence lower the CO_2_ emissions resulting from cement production [3].

Calcium carbide is a dangerous and corrosive waste material obtained as a by-product during the production of acetylene (C_2_H_2_) gas [4,5,6], whereas crumb rubber (CR) is produced from used tires (waste tires) that have been abandoned at mechanic shops and dumpsites [7]. On the other hand, fly ash is a waste material generated from coal-fired electric- and steam-generating plants during coal combustion. In a bid to add value to these waste materials as well as to curb the environmental issues associated with the dumping of these wastes, researchers have been exploring ways to recycle and reuse them efficiently. The use of CR as an aggregate replacement in concrete has been necessitated by the continual increase in the demand for concrete aggregates, with its associated environmental impacts. The environmental impact of using conventional aggregates could lead to erosion, landslides, and natural resource depletion (global warming). The utilization of CR as an aggregate in concrete is eco-friendly and would prevent the burning of used tires, which causes air pollution. Thus, the use of these eco-friendly aggregates in concrete has the potential to mitigate global warming through the prevention of the burning of these waste tires, which releases greenhouse gases into the atmosphere [8].

Research works on the utilization of CCW as well as CR in the production of self-compacting concrete (SCC) have been published [9,10,11].

The durability of SCC is a vital parameter to consider in order to ensure a longer lifespan of the structures constructed with it. This parameter relates to the ability of SCC to retain its original shape, dimension, and quality during its estimated service life when subjected to the toughest weathering conditions, abrasion, and diverse chemical attacks. Tests such as chloride attack/acid attack, sulphate resistance, water absorption, and porosity tests are conducted when assessing the durability of SCC. Several studies have been conducted on the durability of conventional concrete, with a few focused on conventional SCC and very little research conducted on SCC ternary blends. Rajamony Laila et al. [12] investigated the effect of super absorbent polymer (SAP) on the durability and mechanical performance of SCC. They replaced cement using granite Pulver (GP) at 5 to 20% by weight. They used the SAP at 0.1 to 1% by volume fraction of the GP-SCC mixtures. Their results showed that SAP decreased the filling ability and flowability of the SCC. Furthermore, GP increased the early strength development of the SCC at 7 days. The addition of 0.3 and 0.4% SAP significantly improved the flexural, compressive, and splitting tensile strengths of the SCC. In terms of durability, GP improved the resistance of the SCC to acid and sulphate attack. They concluded that up to 15% cement can be replaced by using GP to improve the durability and mechanical properties of the SCC. In another study, Rajamony Laila et al. [13] utilized GP and SAP in conventional concrete and reported similar findings. A study by Surya et al. [14] on the compressive strength of SCC was carried out under elevated temperatures. It was found that as the temperature increases, the compressive strength is decreased. Furthermore, the decrease in the compressive strength was found to be greater at 600 °C and 800 °C, conversely to the effects observed at 200 °C and 400 °C. The study concluded that higher elevated temperature has a negative effect on the compressive strength of SCC. According to Santos et al. [15], an increase in the percentage of recycled coarse aggregate increased chlorine penetration depths. SCC mixes containing 40% recycled coarse aggregate were reported to show better resistance to chloride penetration and acid attack in this study. However, research by Yehia et al. [16] presented low chloride permeability for all mixes, which is an indication of the satisfactory durability of SCC and fiber-reinforced SCC. The low chlorine penetration obtained from their studies was attributed to the high density of the mix. In addition, Babu and Nazeer [17] reported optimum resistance to chloride-ion penetration for SCC ternary blend specimens with 10% silica fume replacement. The results of their studies indicated that there was a high sulphate attack resistance for ternary blended concrete with 5% replacement of silica fumes.

Research carried out by Danish and Ganesh [18] established that the addition of metakaolin and fly ash in SCC mixes reduces water absorption. This could be attributed to the ability of the metakaolin and fly ash to act as fillers by improving the microstructure of the SCC. It was observed that the control mix gave the highest water absorption of 4.84% and 4.04% at 28 and 90 days, respectively, while the incorporation of 15% metakaolin resulted in a lower water absorption of 2.02% and 1.60% at 28 and 90 days, respectively. Hence, the incorporation of metakaolin in the SCC mix resulted in a positive effect of decreasing the water absorption of the mix. Another investigation by Santos et al. [15] showed that the incorporation of a high amount of recycled coarse aggregate in SCC mixes displayed a high initial water absorption level that led to an increase in the permeability values of the mix. The results of the review conducted on the use of waste tire rubber in concrete showed that the water absorption of both SCC and traditional concrete was increased on the addition of waste tire rubber due to the rubber particles’ rough surfaces. It was also speculated that increasing the water/cement ratio will increase water absorption [19].

The addition of admixtures such as metakaolin and fly ash has a positive influence on the porosity in SCC mixes [18]. A concrete mix with an increased porosity network tends to result in a decreased strength of the mix as well as a reduced durability [8,20]. Bayuaji et al. [21] investigated the properties of geopolymer paste produced with CCW and fly ash. The results of their research indicated that samples with the highest level of CCW and fly ash showed the lowest porosity at an age of 56 days. Abdurra’uf et al. [22] also reported an increased porosity in mortar with the increase in the CCW content when used as a cement replacement in mortar. A review conducted by Bušić et al. [19] indicated that an increase in rubber aggregate content in SCC mixtures resulted in an increase in porosity and air content of the mix due to the entrapment of air in the interfacial transition zone between the cement paste and rubber particles.

Furthermore, CCW has been used for soil modification and stabilization [23,24]. Fly ash has also been utilized and recommended by researchers for the partial replacement of cement in concrete [18,25,26]. For the purpose of environmental sustainability, specifically to achieve a reduction in the pollution of the environment and a reduction in the demand for natural materials such as aggregates in concrete, CR has been used to partially replace fine or coarse aggregate in concrete. In SCC, CR has been used to partially replace fine aggregate. Several researchers reported the positive effect of the CR in SCC. For example, Hilal [9] reported an improvement in the fracture energy of SCC with the replacement of up to 5% of fine aggregate with CR, and improved ductility with the replacement of up to 25% of fine aggregate using CR. Najim and Hall [27] also reported a significant improvement in the strain energy of SCC, which resulted in reduced crack mouth open displacement. Additionally, they reported an improvement in ductile behavior, energy absorption, and vibration damping in SCC with the replacement of fine aggregate with CR. Khalil et al. [28] reported an improvement in the impact resistance of SCC when up to 30% of fine aggregate was replaced with CR. In a similar study, Adamu et al. [29] also reported improved impact resistance with the replacement of up to 30% of fine aggregate in concrete. However, the major disadvantage of using CR in concrete and SCC is the reduction in mechanical properties and durability performance. These reductions in mechanical properties and durability are more severe with the increment in CR content, as reported in several studies [9,27,30].

As reported, both fly ash and CR have significant advantages when used in SCC; these include a reduction in the cost of the concrete, greater environmental and material sustainability, and improved waste management. However, their main disadvantages include a reduction in the mechanical properties and durability of SCC due to their negative effects. Therefore, in this study, CCW was used as an additive by weight of cementitious materials to mitigate the negative effect of CR on the durability of SCC and activate the pozzolanic reaction of fly ash at early ages to improve the durability of SCC. Additionally, the use of a combination of the three materials (CCW, CR, and fly ash) to produce an environmentally sustainable SCC is yet to be fully explored. Therefore, the objective of this study was to investigate the durability properties of rubberized SCC containing CCW and fly ash as supplementary cementitious materials in normal and adverse conditions. In this research, water absorption, acid resistance, sulphate resistance, and heat resistance tests were carried out to determine the durability of the blended rubberized SCC.

## 2. Materials and Methods

### 2.1. Materials

**Figure 1:**
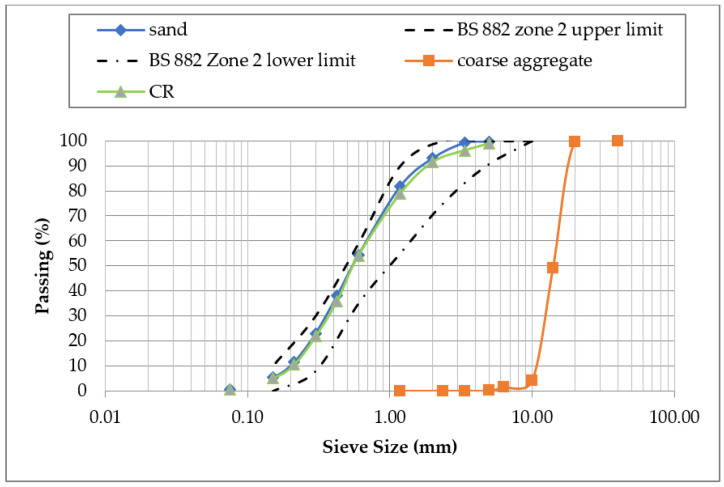
Particle size distribution of coarse aggregate.

Type 1 Ordinary Portland cement with a specific gravity of 3.5, conforming to the guidelines outlined in BS EN 196-6 [31], was used as the main binder material in this study. The cement has chemical properties, which were established using X-ray Fluorescence and are presented in Table 1. Class F fly ash, conforming to the requirements of ASTM C618 [32], was used as a supplementary cementitious material. The properties of the fly ash are also presented in Table 1. The CCW was collected from a commercial welding workshop disposal site in Kano, Nigeria. After that, it was first air dried for about four days and then oven dried for 24 h at a temperature of 110 ± 5 °C to make it completely dry. The CCW was then ground in a grinding machine and sieved through a No. 325 (45 µm) sieve. The particles retained on the sieve were discarded and the ones that passed through were used. The properties of the CCW were then determined using XRF analysis and the results are presented in Table 1. The fine aggregate used was natural river sand, with a specific gravity of 2.63, a bulk density of 1560 kg/m^3^, a water absorption rate of 1.96%, and a mud content of 1.1%. The particle size distribution of the fine aggregate is presented in Figure 1, and belongs to the zone II class based on the grading limit of BS 882 [33]. The CR used had a specific gravity of 0.95, and particle size distribution similar to that of the fine aggregate, as shown in Figure 1. Crushed granite with a maximum size of 19 mm was used as the coarse aggregate. The coarse aggregate had a specific gravity of 2.65, a bulk density of 1450 kg/m^3^, and a water absorption rate of 0.94%; its particle size distribution is shown in Figure 1. A high-range water-reducing admixture was added by weight of cementitious materials to achieve the self-compaction. The dosage of the superplasticizer was used based on the manufacturer’s specification of 2 to 15 fl.oz/cwt of cementitious materials.

### 2.2. Mix Proportioning

To examine the effect of CR and CCW on the durability performance of SCC under normal and adverse conditions, mixes were prepared by partially replacing fine aggregate with CR at 0%, 10%, and 20% using a volume replacement method, and CCW was used as a partial replacement to cement at 0%, 5%, and 10% by means of a volume replacement method. To further study the effect of fly ash on the durability of SCC blends, fly ash was used to replace 40% cement, with CR replacing fine aggregate at 0%, 10%, and 20% by volume, and CCW replacing cement at 0%, 5%, and 10% by volume. A total of eighteen mixes were prepared, as presented in Table 2. The mixes were tested for acid attack, salt attack, elevated temperature, and water absorption. Each mix was assigned a unique ID; for example, mix M0CR0C is the control mix with 0% CR and 0% CCW, and mix M0CR40F0C is the mix with 0% CR, 40% fly ash, and 0% CCW. Mix M10CR5C is the mix with 10% CR and 5% CCW, and mix M20CR40F10C is the mix with 10% CR, 40% fly ash, and 10% CCW. The superplasticizer dosage used was 1.5% by weight of binder materials.

### 2.3. Casting of Specimen

The batching, mixing, and sampling of the fresh concrete was carried out in accordance with the guidelines outlined in BS 1881-125 [34]. A rotating pan mixer was used for the mixing of the fresh concrete. Before mixing, it was ensured that the cement, fly ash, and CCW were completely dried and free from lumps, and that the aggregates were in saturated surface dry condition to avoid the absorption of mixing water. The fine aggregate was first poured into the mixer, followed by the cement, fly ash, and CCW. They were then allowed to mix for about 30 s. After that, the coarse aggregate and half of the mixing water was added, and the mixing continued. The superplasticizer was mixed with the other half of the mixing water and was poured gently to the concrete in the mixer. The mixing was continued for about 2 min until a completely homogenous paste was achieved. The mixing was carried out at a room temperature of 20 ± 5 °C and controlled relative humidity. Immediately after mixing, the fresh concrete was casted into the molds. Prior to casting, the molds were cleaned and oiled for ease of demolding. The samples were then air dried for 24 h at room temperature before demolding. After demolding, the samples were fully immersed in a clean water for the curing periods prior to testing.

The durability test was carried out in accordance with ASTM C642 [35]. The 100 mm cube specimen, after curing for 28 days, was weighed before being dipped in acid, and was reweighed after 3, 7, and 28 days. H_2_SO_4_ was used as the acidic medium while MgSO_4_ was used as the salt medium. The choice of these chemicals was made based on its high reactivity compared to other acids and salt; therefore, it can be considered when designing structures for worst-case scenarios in terms of exposure to high-acid or salt-prone environments. For each mix, three samples were tested, and the mean values were recorded. Cubes were weighed and then subjected to heat for one hour each at a temperature of 100 °C, 200 °C, 300 °C, 400 °C, and 500 °C, respectively, allowed to cool, and then reweighed before being subjected to a compressive strength test. The compressive strength test was carried out on the 100 mm cube samples in accordance with BS EN 12390-3 [36] using a universal testing machine of 2000 kN capacity. Water absorption of the 100 mm cube specimens was also tested after a 28-day curing period in accordance with the method outlined in ASTM C642 [35].

## 3. Results and Discussions

### 3.1. Durability of SCC on Immersion in Acid Media

**Figure 2:**
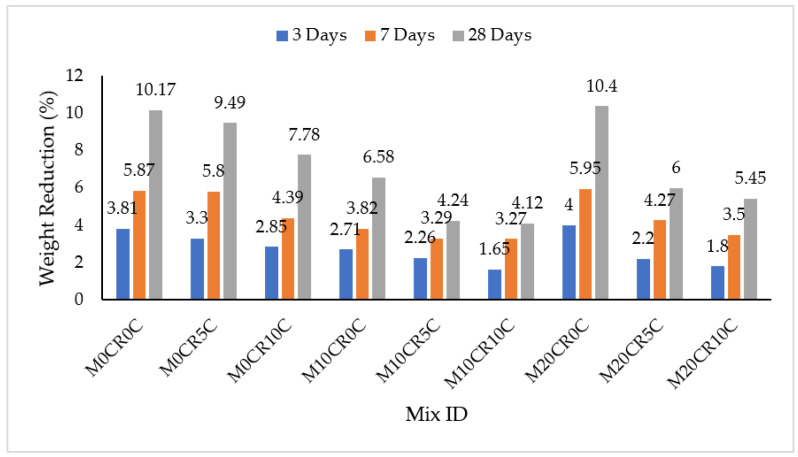
Immersion periods in H_2_SO_4_ for SCC mixes containing CR and CCW.

**Figure 3:**
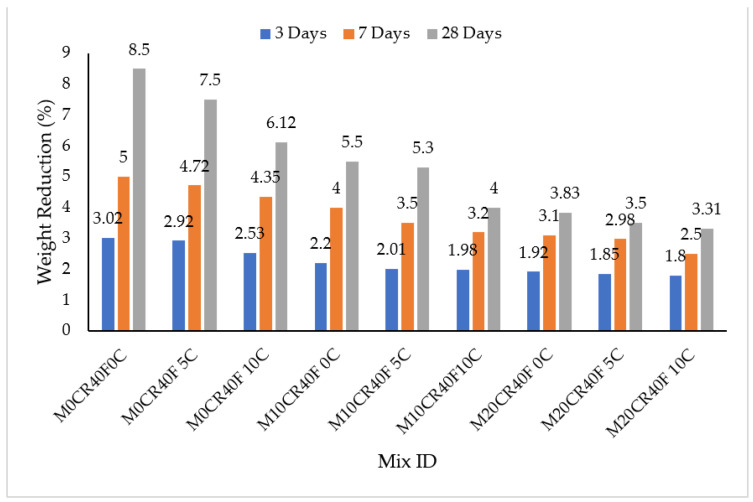
Immersion periods in H_2_SO_4_ for SCC mixes containing fly ash, CR, and CCW.

The effect of acid attack on SCC mixes was measured by immersion in H_2_SO_4_ concentration and the results are presented in Figure 2 and Figure 3. It can clearly be seen that sulphate decreased the weight of all the SCC mixes, as presented in Figure 2 and Figure 3. The decrease in weight (%) can be attributed to the large amount of CaO in the CCW and Portland cement and its hydration product Ca(OH)_2_, which is primarily responsible for the poor resistance of SCC exposed to acidic attack [37]. The weights of all of the SCC mixes were reduced with the increasing of the immersion period in H_2_SO_4_. This was a result of continuous attack by the sulphate over time. However, the attack by sulphate was reduced for mixes containing fly ash, as shown in Figure 3. At 28-day immersion age, the M10CR10C mix had the greatest resistance to acid attack for the mixes not containing fly ash, while the M20CR40F10C mix had the greatest resistance to acid attacks for mixes with fly ash, with a 16% reduction in acid penetration upon incorporation of HVFA to the mix. On the other hand, the M20CR0C mix had the least resistance to acid attack for mixes containing no fly ash, while the M0CR40F0C mix had the least resistance to acid attack for mixes with a 40% replacement of cement with fly ash, showing a 23% reduction in acid penetration upon incorporation of fly ash to the mix.

The reason for the improvement could be attributed to the pozzolanic reactivity of fly ash in the mix. In addition, the better finishing surface and the minimal voids on the concrete surface of the specimens containing fly ash led to lower penetration of the acid solution into the interior of the concrete and improved its resistance against acid attack [38]. This observation is similar to that of Dhiyaneshwaran et al. [37]. In contrast, attack by sulphate was increased with the increment in the percentage replacement of fine aggregate with CR for mixes without fly ash. This was due to the presence of the micro voids in the samples, which gave room for more penetration of the immersed media. The higher the quantity of the content of the CR, the greater the number of micro voids present, giving rise to more expansions. This is similar to the finding of Xu et al. [39]. It can be seen that the addition of fly ash corrected the sulphate attack associated with the replacement of aggregate with CR for the mix with the least penetration, namely M20CR40F10C.

The addition of CCW to SCC mixes with and without fly ash decreased its resistance to acid attack at all ages of curing, as measured by immersion in H_2_SO_4_ solution. This can be seen in Figure 2 and Figure 3, where mixes containing higher CCW exhibit lower weight reduction. For instance, compared to the control mix (M0CR0C), the weight losses for mixes M0CR5C and M0CR10C were lower by 6.69% and 23.50% at 28 days, respectively. For mixes with fly ash, the M0CR40F5C and M0C40F10C were lower by 11.76% and 28%, respectively, at 28 days. This weight reduction due to CCW was attributed to the high amount of CaO in the CCW, thereby providing excess Ca(OH)_2_ in the cement matrix. These calcium compounds are readily attacked by the H_2_SO_4_, which is a strong acid [40]. Similar findings have been reported by Sata et al. [41] and Hanjitsuwan et al. [40], namely a decreased acid resistance of concrete containing CCW as a binder material. For mixes containing 40% fly ash, the reductions in acid attack (sulphate resistance) of the SCC mixes were lower compared to the mixes without fly ash. This can be observed by comparing the values of weight reductions in Figure 2 and Figure 3. This is attributable to the fact that fly ash decreased the calcium content in the cement matrix via a pozzolanic reaction between the SiO_2_ from the fly ash with Ca(OH)_2_ from the CCW to generate more C-S-H gels.

### 3.2. Immersion in Salt Media

**Figure 4:**
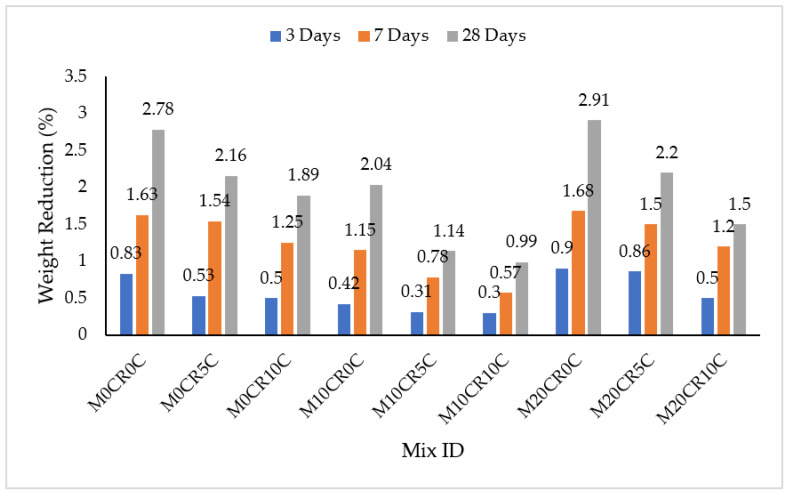
Immersion periods in MgSO_4_ for SCC mixes containing CR and CCW.

**Figure 5:**
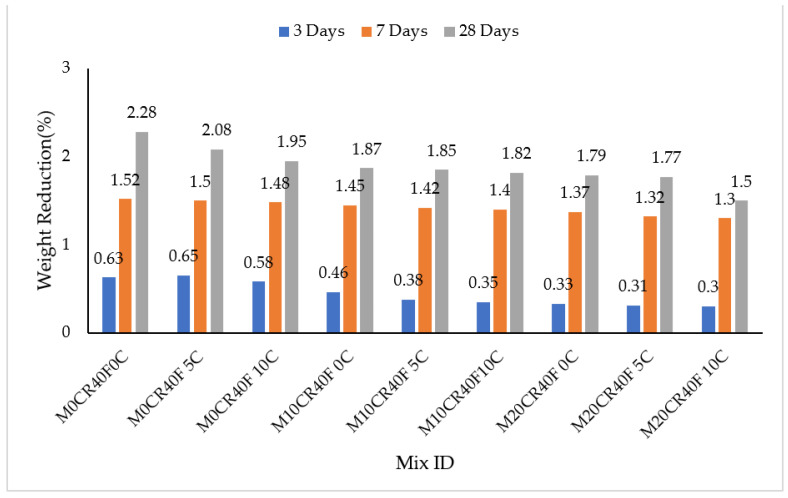
Immersion periods in MgSO_4_ for SCC mixes containing fly ash, CR, and CCW.

The effect of salt attack on the SCC mixes was determined by immersing the SCC in a 5% concentration of MgSO_4_, and the results are presented in Figure 4 and Figure 5. The weight of all the SCC blends was reduced with an increase in immersion age in the MgSO_4_. This was a result of a continuous attack by the sulphate with time, but the rate of the attack was slower compared to the attack by H_2_SO_4_. On the other hand, the durability performance of the SCC mixes with or without fly ash in terms of salt resistance decreased with the increment in the partial replacement of fine aggregate using CR. For the mix without fly ash, namely mix M10CR0C, there were reductions in weight by 49.40%, 29.45%, and 26.62% at 3, 7, and 28 days, respectively, compared to the control mix (M0CR0C). For mixes with fly ash, the weight reductions of mix M10CR40F0C were lower by 26.98%, 4.61%, and 17.98% at 3, 7, and 28 days, respectively, compared to mix M0CR40F0C. Similarly for mix M20CR40F0C, the weight losses were lower than that of M0CR40F0C by 47.62%, 9.87%, and 21.49% at 3, 7, and 28 days, respectively. This reduction in salt resistance due to CR was attributed to increased porosity in the hardened cement matrix and the weak interfacial transition zone caused by the CR in the cement matrix. This further instigated the formation of internal pressure by the sulfate-related crystal growth, i.e., the formation of ettringite and gypsum due to the MgSO_4_; this adversely affected the integrity of the SCC through the further formation of internal cracks, and the escalation of the pores, which was caused by the CR. This consequently resulted in decreased resistance to salt attack (MgSO_4_) [42].

The addition of CCW to SCC mixes without fly ash further decreased the weight loss due to immersion in MgSO_4_ at all ages of curing. At 28 days, the weight losses of mixes M0CR5C, M0CR10C, and M20CR10C were lower by 22.3%, 32.01%, and 46.04%, respectively, in comparison to the control mix (M0CR0C). Similarly, for some mixes with fly ash, the weight reduction due to immersion in MgSO_4_ decreased with the addition of CCW. At 28 days, the weight losses for mixes M0CR40F10C, M10CR40F5C, and M10CR40F10C were lower by 14.47%, 18.86%, 34.21%. The reduction in MgSO_4_ resistance caused by CCW can be attributed to the high Ca(OH)_2_ generated by the CCW in the cement matrix. This Ca(OH)_2_ reacts with the MgSO_4_ to liberate the hydration of C_3_S and C_2_S in the cement, thereby instigating gypsum formation, leading to magnesium-gypsum attack on the C-S-H formed during cement hydration, thus causing deterioration [43]. By comparing Figure 4 with Figure 5, it can be observed that the resistance of SCC to salt attack, i.e., immersion in MgSO_4_, was higher for mixes with fly ash compared to mixes without fly ash. This can be attributed to the pozzolanic reaction of fly ash with CCW, i.e., SiO_2_ from fly ash reacting with the excess Ca(OH)_2_ produced from CCW to generate more C-S-H gels. This consequently reduced the amount of Ca(OH)_2_ that reacted with MgSO_4_ to form magnesium based-gypsum, which caused deterioration of the cement matrix [43].

### 3.3. Effect of Elevated Temperature on SCC Mixes

**Figure 6:**
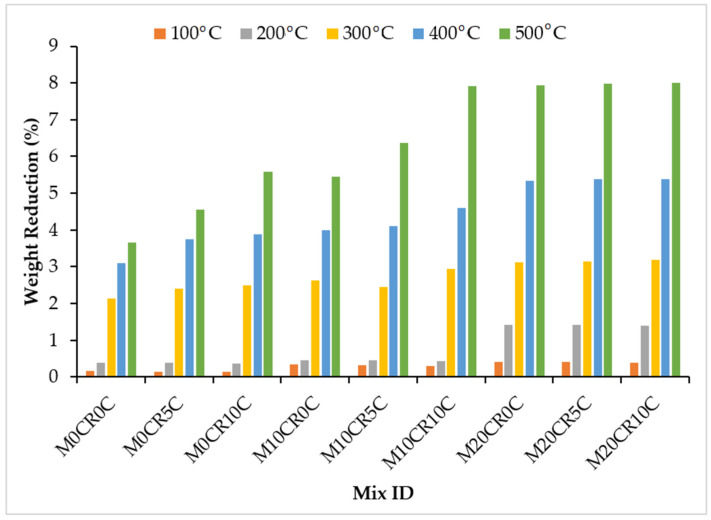
Effects of elevated temperature on weight of SCC mixes containing CR and CCW.

**Figure 7:**
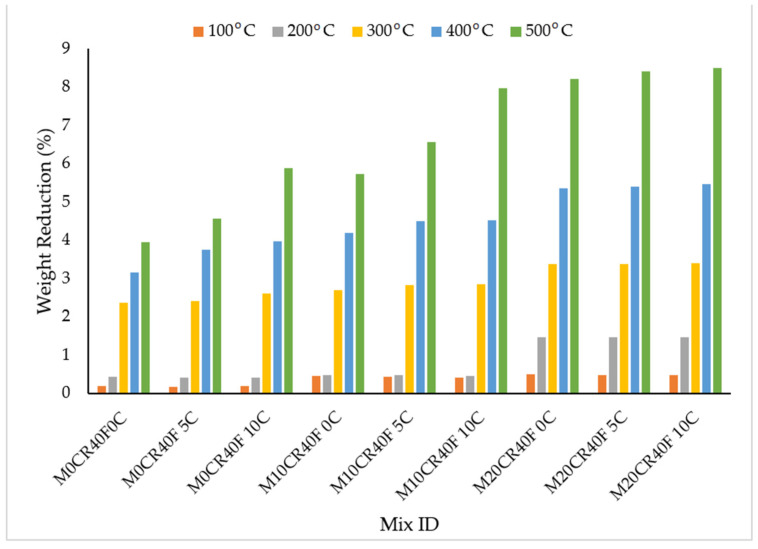
Effects of elevated temperature on weight of SCC mixes containing fly ash, CR, and CCW.

Figure 6 and Figure 7 shows the percentage weight reduction in the SCC mixes at varied temperatures. These figures show that the SCC blends lost more of their weight as the CR content was increased. This observation was noticed as the temperature increased, as shown in the aforementioned figures. The reduction in weight could be a result of spalling caused by internal water pressure at about 300 °C and during the dehydration of hydrated calcium silicate hydrate, which is known to increase internal stresses and induces micro-cracks [44]. The decrease in weight could also have resulted from the expulsion of the excess pore water in the SCC mix [45]. Generally, the addition of 40% optimum fly ash yielded a further decrease in the weight of the mixes by 6%, when compared with the mix without fly ash (M20CR10C) at 500 °C and the M20CR40F10C mix for mixes with fly ash. This confirms a similar result obtained by Fawzy et al. [46] after heating at 200 °C and below.

The weight reduction was reduced slightly with the increase in the addition of CCW due to the reaction of CCW with the free lime, which produced more CSH and CAH. The samples exhibited no sign of explosive spalling, but the test samples showed cracks on their surface, which were increased as the temperature increased due to water evaporation, drying shrinkage, and thermal expansion that mounted stress on the concrete, resulting in cracks [22].

**Figure 8:**
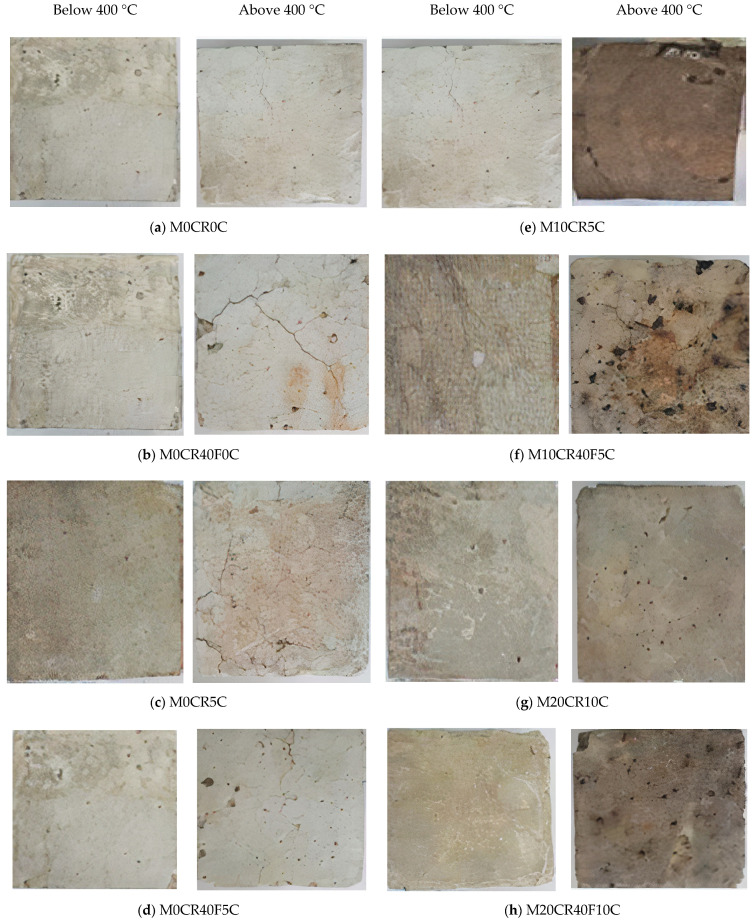
Samples’ color changes after heating at elevated temperature. (**a**) M0CR0C (**b**) M0CR40F0C (**c**) M0CR5C (**d**) M0CR40F5C (**e**) M10CR5C (**f**) M10CR40F5C (**g**) M20CR10C (**h**) M20CR40F10C.

In addition, the color of the SCC control mix became brighter because of the water loss at high temperatures, while the mix containing CR was changed from light grey to brownish-black due to the dispersion of carbon black, which is a part of the rubber tires, with the increase in temperature from 400 °C to 500 °C. Certain colors correspond with a specific temperature range, which is an important indicator of the maximum temperature that the structure can be exposed to. At temperatures below 400 °C, the concrete color did not change noticeably, as shown in Figure 8. This observation is similar to the findings of Fawzy et al. [46].

**Figure 9:**
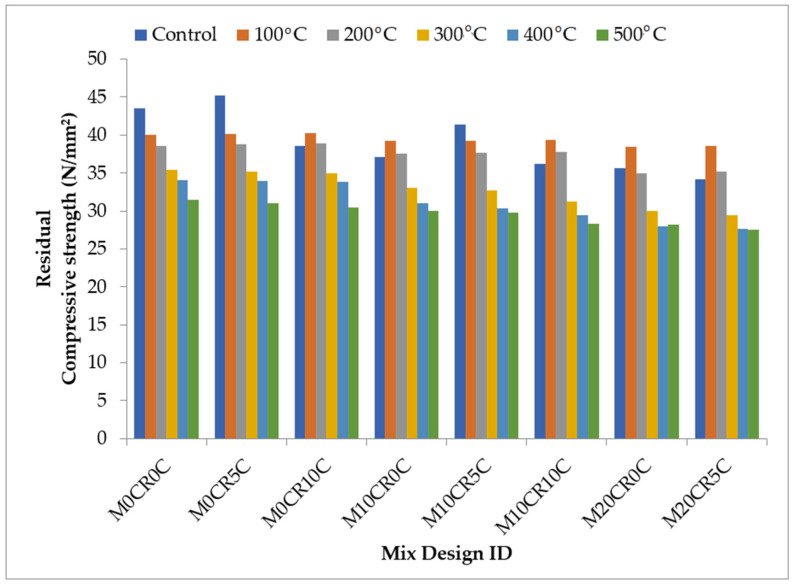
Effect of elevated temperature on the compressive strength of SCC mixes containing CR and CCW.

**Figure 10:**
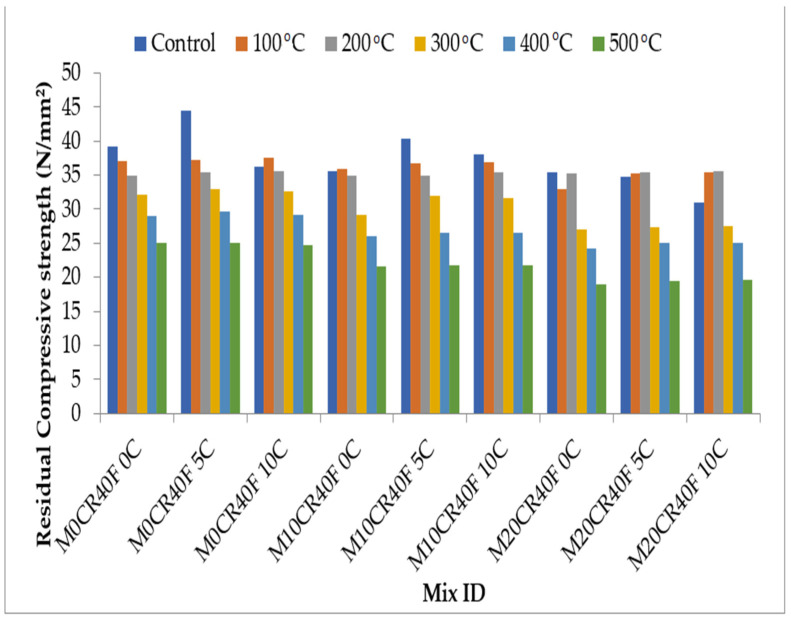
Effect of elevated temperature on the compressive strength of SCC mixes containing fly ash, CR, and CCW.

The compressive strengths of the various replacements when subjected to elevated temperatures of 100, 200, 300, 400, and 500 °C are graphically represented in Figure 9 and Figure 10. The compressive strength of SCC mixes was decreased with the increase in the temperature. A slight increase in the compressive strength was observed for all mixes and their replicas containing fly ash at control temperature (27 °C) except for the M10CR0C, M10CR0C, M10CR10C, M20CR0C, and M20CR5C mixes, which experienced increases in compressive strength at 100 °C before a further decrease. This was due to the reduction in the calcium hydroxide and un-hydrated area fraction aided by autoclaving (heat healing effect), which enhanced the microstructure [47]. The highest loss in compressive strength was observed at the temperature between 300 and 400 °C due to the calcium silicate hydrate (C-S-H). The incorporation of CR to the mix also reduced the rate of compressive strength loss because the melted rubber particle at 200 °C left space for water vapor to escape and, thus, helped to release the pore pressure. This outcome is similar to the findings of Huang et al. [48]. Generally, it can be observed that CCW slightly increased the compressive strength up to 200 °C and then decreased it after that. This increase was a result of the reaction of CCW with the free lime to produce more CSH and CAH, which deposited in the pore system, and also occurred because of the higher volume of CSH and CAH phases formed and the reduction in Ca(OH)_2_ content.

### 3.4. Water Absorption

**Figure 11:**
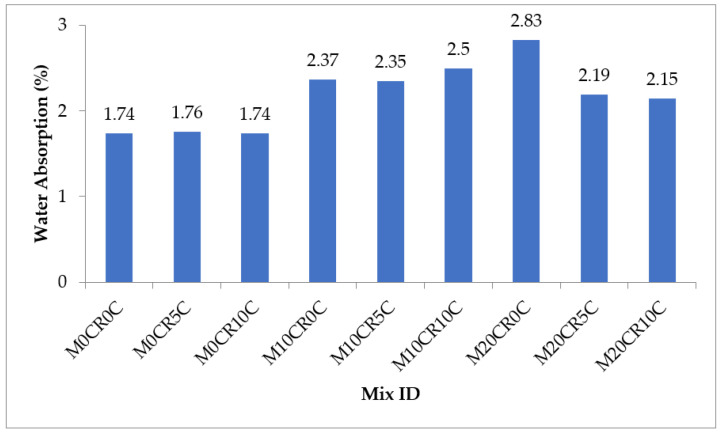
Water absorption capacity of SCC mixes containing CR and CCW.

**Figure 12:**
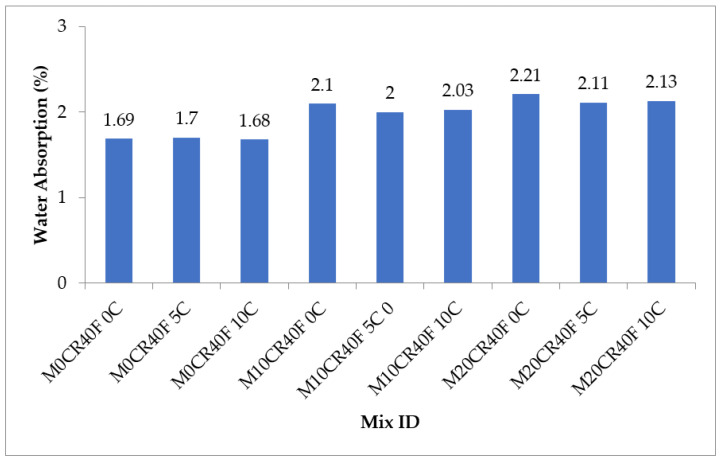
Water absorption of SCC mixes containing fly ash, CR, and CCW.

Figure 11 and Figure 12 show the water absorption capacity of the various SCC mixes. The water absorption of the SCC mixes increased with the increment in the partial replacement of fine aggregate with CR for the mixes with and without fly ash. For mixes without fly ash, the water absorption of mixes M10CR0C and M20R0C was higher, by 36.21% and 62.64%, respectively, compared to mix M0CR0C (control mix). Similarly, for mixes with fly ash, the water absorption of mixes M10CR40F0C and M20CR40F0C was lower, by 24.26% and 30.77%, respectively, compared to mix M0CR40F0C. The reduction in water absorption due to CR incorporation can be attributed to the hydrophobic nature of CR, causing it to entrap air on its surface during mixing. When the concrete dried up, the entrapped air left excess voids in the cement matrix, thereby increasing porosity and, hence, leading to higher water absorption [7,8]. The addition of CCW slightly increased the water absorption of mixes without fly ash. For instance, the water absorption of mix M0CR5C was slightly higher than that of the control (by 1.15%), while mix M0CR10C had the same water absorption value as the control mix (M0CR0C). Furthermore, the addition of CCW partially mitigated the negative effect of CR on the water absorption of the SCC mixes with or without fly ash. This can be observed by comparing the water absorption value of M20CR0C (2.83%) with that of M20CR5C (2.19%) and M20CR10C (2.15%), and that of mix M20CR40F0C (2.21%) with M20CR40F5C (2.11%) and M20CR40F10C (2.13%). The reduction in water absorption due to the addition of CCW can be attributed to its finer sizes, thereby acting as a filler material and filling the pores created by the CR and, hence, reducing the water absorption. Another reason could be the higher reactivity of CCW, thereby allowing it to react with cement hydration products to form more C-S-H gels, which filled the nanopores in the cement matrix and, hence, reduced the water absorption [4,6]. By comparing Figure 11 with Figure 12, it can be seen that for the SCC with fly ash, mixes with same amount of CR and CCW had lower water absorption values compared to the SCC mixes without fly ash with the same amount CR and CCW. This was due to the pozzolanic reaction between the SiO_2_ from fly ash and Ca(OH)_2_ from CCW to generate excess C-S-H gels, which filled up the pores in the cement matrix and the decreased water content, similarly to the in the SCC without fly ash. Therefore, it can be concluded that the incorporation of CCW and fly ash reduces the water absorption due to the refined pore system created by fly ash and the CCW microstructure. Overall, the water absorption of the SCC blends containing the replacements in the right proportion resulted in a more durable SCC. This is in line with the research conducted by Mohammed and Adamu [8].

## 4. Conclusions

In this study, the effect of the partial replacement of fine aggregate with CR, and of cement with CCW, on the durability performance of SCC in both normal and adverse conditions was investigated. Furthermore, the effect of CR as an aggregate replacement, and of CCW as a cement replacement, on a blend of SCC made produced 40% fly ash as a supplementary cementitious material was evaluated. Based on the experimental results and analysis, the following conclusions can be drawn.

(1) The resistance of the SCC against acid attack, as measured by immersion in H_2_SO_4_, and the resistance against salt medium, as measured by immersion in MgSO_4_, all improved with the use of fly ash and CCW as supplementary cementitious materials (SCM).
(2) The partial replacement of fine aggregate with CR in SCC mixes significantly reduced its resistance against acid and salt attacks. This negative effect was more severe in the SCC mixes without fly ash and CCW.
(3) The resistance of the SCC mixes against heat decreased the replacement of 40% cement with fly ash beyond a temperature of 200 °C. Furthermore, the heat resistance of the SCC mixes decreased with the increase in the partial replacement of fine aggregate with CR, and the use of CCW as SCM.
(4) The optimum temperature for the blends was found to be 400 °C; however, the water absorption was decreased with the increasing of the fly ash content and was increased with the increasing of the CR content, but with a rapid increase beyond 10% addition of CR.
(5) Fly ash is recommended for use as a SCM to improve the resistance of SCC mixes containing CR as a partial replacement of fine aggregates for use in acidic and salty environments.

## Figures and Tables

**Table 1:** Chemical properties of materials.

Oxide	Chemical Compositions (%)		
	Cement	CCW	Fly Ash
SiO_2_	12.00	1.10	52.06
Al_2_O_3_	3.01	0.04	30.48
Fe_2_O_3_	4.11	0.50	4.47
CaO	74.03	96.46	5.62
MgO	1.30	-	1.32
K_2_O	1.28	0.45	1.54
Na_2_O	0.19	0.01	0.99
SO_3_	2.07	0.29	2.1
Loss of Ignition	1.02	2.24	0.95
Specific Gravity	3.15	2.22	2.27
Specific Surface Area (m^2^/g)	325	295	290

**Table 2:** Mix Proportioning.

Mix ID	Cement (kg/m^3^)	CCW (kg/m^3^)	Fly Ash (kg/m^3^)	Fine Aggregate (kg/m^3^)	CR (kg/m^3^)	Coarse Aggregate (kg/m^3^)	Water (kg/m^3^)	SP (kg/m^3^)	W/B
M0CR0C	520	0	0	880	0	850	192.4	7.80	0.37
M0CR40F0C	312	0	149.89	880	0	850	192.4	6.93	0.42
M0CR5C	494	18.32	0	880	0.00	850	192.4	7.68	0.38
M0CR40F5C	286	18.32	149.89	880	0.00	850	192.4	6.81	0.42
M0CR10C	468	36.65	0	880	0.00	850	192.4	7.59	0.38
M0CR40F10C	260	36.65	149.89	880	0.00	850	192.4	6.70	0.43
M10CR0C	520	0.00	0	792	38.25	850	192.4	7.80	0.37
M10CR40F0C	312	0.00	149.89	792	38.25	850	192.4	6.93	0.42
M10CR5C	494	18.32	0	792	38.25	850	192.4	7.68	0.38
M10CR40F5C	286	18.32	149.89	792	38.25	850	192.4	6.81	0.42
M10CR10C	468	36.65	0	792	38.25	850	192.4	7.59	0.38
M10CR40F10C	260	36.65	149.89	792	38.25	850	192.4	6.70	0.43
M20CR0C	520	0.00	0	704	76.49	850	192.4	7.80	0.37
M20CR40F0C	312	0.00	149.89	704	76.49	850	192.4	6.93	0.42
M20CR5C	494	18.32	0	704	76.49	850	192.4	7.68	0.42
M20CR40F5C	286	18.32	149.89	704	76.49	850	192.4	6.81	0.42
M20CR10C	468	36.65	0	704	76.49	850	192.4	7.59	0.38
M20CR40F10C	260	36.65	149.89	704	76.49	850	192.4	6.70	0.43

## Data Availability

The data presented in this study are available on request from the corresponding author.
